# Pulmonary Function Tests in Emergency Department Pediatric Patients with Acute Wheezing/Asthma Exacerbation

**DOI:** 10.1155/2012/724139

**Published:** 2012-12-10

**Authors:** Kathryn Giordano, Elena Rodriguez, Nicole Green, Milena Armani, Joan Richards, Thomas H. Shaffer, Magdy W. Attia

**Affiliations:** ^1^Department of Emergency Medicine, Nemours/Alfred I. duPont Hospital for Children, Wilmington, DE 19803, USA; ^2^Nemours Research Lung Center, Nemours/Alfred I. duPont Hospital for Children, Wilmington, DE 19803, USA; ^3^Department of Respiratory Care, Nemours/Alfred I. duPont Hospital for Children, Wilmington, DE 19803, USA; ^4^Department of Pediatrics, Jefferson Medical College, Philadelphia, PA 19107, USA; ^5^Departments of Physiology and Pediatrics, Temple University School of Medicine, Philadelphia, PA 19140, USA

## Abstract

*Background*. Pulmonary function tests (PFT) have been developed to analyze tidal breathing in patients who are minimally cooperative due to age and respiratory status. This study used tidal breathing tests in the ED to measure asthma severity. *Design/Method*. A prospective pilot study in pediatric patients (3 to 18 yrs) with asthma/wheezing was conducted in an ED setting using respiratory inductance plethysmography and pneumotachography. The main outcome measures were testing feasibility, compliance, and predictive value for admission versus discharge. *Results*. Forty patients were studied, of which, 14 (35%) were admitted. Fifty-five percent of the patients were classified as a mild-intermittent asthmatic, 30% were mild-persistent asthmatics, 12.5% were moderate-persistent asthmatics, and 2.5% were severe-persistent. Heart rate was higher in admitted patients as was labored breathing index, phase angle, and asthma score. *Conclusions*. Tidal breathing tests provide feasible, objective assessment of patient status in the enrolled age group and may assist in the evaluation of acute asthma exacerbation in the ED. Our results demonstrate that PFT measurements, in addition to asthma scores, may be useful in indicating the severity of wheezing/asthma and the need for admission.

## 1. Introduction

Asthma and other childhood wheezing disorders represent a significant percentage of pediatric emergency department (ED) visits [1]. Management decisions are largely based on exam findings, vital signs, and pulse oximetry [2]. The child and parents' perception of the clinical symptoms are important tools in the guidelines for assessing asthma severity. Dyspnea, cough, wheezing, and exercise intolerance are helpful in making clinical decisions, but it is well known that neither the child nor the parent accurately report these symptoms. Their perceptions often underestimate the severity of the disease [3].

Preschool children (<5 years of age) make up a large percentage of the asthmatics presenting to the pediatric ED; however, because of their inability to cooperate with spirometry or peak flow, they are frequently excluded from research studies. An estimated 40% of children aged 6 to 9 years and 25% of children under 10 years would likely be excluded from participation in trials if spirometry was the objective measure of asthma severity [3, 4]. Pulmonary function tests (PFT) have been developed to analyze tidal breathing in patients who are minimally cooperative due to age or clinical condition (urgency associated with the ED). These include respiratory inductance plethysmography (RIP) and pneumotachography (PT), which have been used extensively in the minimally cooperative neonate and pediatric populations [5–12].

In the present pilot study, we hypothesized that PFT in the ED would provide a predictive value associated with disposition (admission versus discharge) to aid in resource utilization. Since these measurements have not been utilized in this setting, we set out to determine the feasibility of performing these measurements in this setting, the compliance of patients with the evaluation techniques, and the post-hoc predictive value of the results (clinicians were initially blinded to the PFT results at the time of admission). Finally, we were interested in an assessment tool employing tidal breathing analysis that suggests the severity of the wheezing exacerbation. Thus, we are speculating that this objective diagnostic approach may be used in the future to determine from the moment of triage the likelihood that a given patient would require a prolonged ED visit. A decision regarding disposition could then be made early, decreasing the patient's ED length of stay (LOS).

## 2. Methods and Materials

### 2.1. Patients and Protocol

We conducted a prospective observational study of 40 patients (aged 3 to 18 years) who presented to the ED with the chief complaint of wheezing or with an asthma exacerbation. The study was reviewed and approved by our Institutional Review Board, and we obtained written, informed parental consent in all cases. Only those patients who were determined to be in mild-to-moderate respiratory distress based on the Emergency Severity Index (ESI v.4) criteria were eligible for enrollment. The ESI v.4 is a five-level triage system based on resource utilization. It is widely used among both adult and pediatric EDs [13]. Exclusion criteria were severe respiratory distress warranting immediate intervention (ESI levels 1 and 2), underlying lung disease (including but not limited to cystic fibrosis or spinal muscular atrophy), and age less than 3 years or greater than 18 years. Once identified by the coinvestigators, the parent/guardian provided consent for enrollment and assent was obtained for patients 7 years and older. The patient then completed an asthma severity questionnaire to give insight to their disease. 

Pulmonary function tests were obtained at baseline, prior to implementing standard of care treatment (i.e., protocol-driven care for asthmatic patients). Tests included RIP and inductance bands. We also evaluated the objective monitoring of real tidal volume, respiratory rate, flow patterns, and end-tidal CO_2_ by tidal breathing analysis with PT. Below is a detailed explanation of these two noninvasive tidal breathing techniques. 

The prescribed clinical treatment was at the discretion of the treating physician. For moderate asthma exacerbation, the treatment regimen includes one nebulized albuterol (dose of 0.15 mg/kg/dose, max 5 mg) and ipratropium bromide (dose of 250 mcg for patients less than 20 kg or 500 mcg for patients greater than 20 kg) followed by either one hour of continuous albuterol nebulization or two additional nebulized albuterol and ipratropium bromide treatments 15 minutes apart with oral corticosteroids (dose of 2 mg/kg, max 60 mg). Mild asthma exacerbations occasionally were treated more conservatively. At times, only one nebulized albuterol treatment was needed with or without oral corticosteroids. The PFT were therefore performed before the initial treatment was prescribed.

The treating physician was blinded to the results of the PFT measurements. The clinical decisions were based on the standards of care that are currently used in the ED, including vital signs (respiratory rate, heart rate, transcutaneous oxygen saturation [Masimo, Irvine, CA]) and clinical appearance. An asthma score was assigned to each patient. This score was calculated as follows: respiratory rate based on age (less than 7 years: 0: 0–30, 1: 31–45, 2: 46–60, 3: >60; greater than 7 years: 0: 0–20, 1: 21–35, 2: 36–50, 3: >50), wheezing (0: absent, 1: expiratory only, 2: expiratory, and inspiratory, 3: audible without stethoscope), and degree of retractions (0: none, 1: mild, 2: moderate, 3: severe) [4, 14].

### 2.2. PFT Methods

PFT methods included RIP using the SomnoStarPT Unit (Sensormedics, Yorba Linda, CA) and inductance bands (RespiBands Plus; VIASYS Respiratory Care, Yorba Linda, CA). We also evaluated the objective monitoring of real tidal volume, respiratory rate, flow patterns, and end-tidal CO_2_ by tidal breathing analysis with PT. For PT, we used a pediatric respiratory profile monitor (CO2SMO Plus; Novametrix, Wallingford, CT). Initially, patients underwent RIP, in which the relationship between thoracic and abdominal contributions to the respiratory effort was assessed (7–9). Bands containing inductive coils were carefully placed around the rib cage at the level of the axillae and around the abdomen mid-way between the xiphisternal junction and the umbilicus. *We used a noncalibrated RIP method for phase and synchrony evaluations and used an abbreviated two-point RIP-tidal breathing calibration for evaluation of labored breathing index (LBI). *The Respitrace device was used to construct Lissajous loops and for calculation of phase angle between the rib cage and abdominal movement associated with respiration (10, 15, 16). Reported phase angle measurements and other indices of asynchrony were based on the average of at least 10 uniform Lissajous loops. The signals from the RIP bands around the rib cage and the abdomen were treated mathematically as sine waves. The phase angle was then calculated with RespiEvents software 5.2 (NIMS, Miami, FL). This parameter and other thoracoabdominal markers express the degree to which chest and abdominal excursion are out of phase. Normally, the rib cage moves outward during inspiration completely in phase with the outward movement of the abdomen (in phase). With progressive increase in the work of breathing, like in airflow obstruction, the rib cage lags behind abdominal movement (out of phase) becoming asynchronous [10].

Recordings were made with the patient in the sitting position. During the test, raw signals and Konno-Mead (Lissajous) loops were monitored to ensure adequate signal quality and to select suitable breathing sequences for analysis with RespiEvents. In this study, we analyzed phase angle (the phase delay between the thoracic and abdominal excursions, or the degree of thoracoabdominal asynchrony [TAA]), phase relation during total breath (PhRTB%), and LBI. 

Pulmonary breathing patterns were assessed using a commercially available neonatal/pediatric pulmonary monitoring system. Airway flow and volume were simultaneously measured over time at the airway opening using a pneumotachometer via face mask; mask integrity was monitored for leaks (less than 10% tidal volume change throughout each breath) and a constant tidal volume breathing frequency history was observed for at least 10 breaths (7–9). Airflow was measured with a low dead space volume pneumotachometer and integrated pressure transducer. Respiratory volumes were determined by integrating time and flow signals. Minute ventilation, respiratory rate, peak expiratory flow, and end-tidal CO_2_ were calculated by the algorithms incorporated into the monitoring system unit. Flow and volume data, as well as the calculated parameters, were recorded using a software package that interfaces with the pulmonary monitoring unit (Analysis Plus, Novametrix) [11]. Flow and CO_2_ graphs were analyzed in real time and after each measurement. Parameters related to timing of peak tidal expiratory flow were extracted from the tidal-flow-volume loops. Flow, volume, and pressure-volume loops as well as CO_2_ graphs are excellent tools for assessing airway obstruction and response to bronchodilator therapy. As an additional safety measurement, the transcutaneous saturation of oxygen (SpO_2_) was monitored simultaneously, as per ED asthma care protocol, with a small sensor on the finger of the patient. 

Tidal breathing measurements required the child to breathe through a mask for 15–30 seconds; the recordings started when we detected a steady and natural respiratory pattern. Based on previous experience, this technique allows repeatability of the study and the mean of the data can then be analyzed with confidence (9). For example, in a patient with a moderate asthma exacerbation and a respiratory rate of approximately 42 breaths per minute, this reading can be obtained in less than 15 seconds. The data of interest included time to peak tidal expiratory flow (*t*_PTEF_), volume at peak tidal expiratory flow (*V*_PTEF_), total expiratory time (*t*_*E*_), expired tidal volume (*V*_*E*_), and the ratios *t*_PTEF_/*t*_*E*_ and *V*_PTEF_/*V*_*E*_ [5].

### 2.3. Statistics

Although this was a feasibility pilot study, we calculated a sample size. We did so with the assumption that the proportion of positive in the population is 0.25 (25% asthma admission rate; internal ED quality assurance data). The effect size was selected as the smallest effect that would be important to detect and was clinically reasonable: 0.2 (based on sensitivity of 80% for the PFT). Alpha was set at 0.05. Data analysis was performed with SPSS 17.0 (Chicago, IL) and graphs were created with GraphPad Prism 5 (GraphPad Software, La Jolla, CA). Quantitative variables were summarized using mean and standard deviation, and categorical variables were summarized using frequencies and percentages. A two-sample *t*-test was used to compare the mean the of quantitative variables, and a chi-square test was used to compare the distribution of categorical variables. A one-tailed *t*-test was chosen to analyze PFT data since normative values are known for the data collected. It is understood that with increased work of breathing indices, the values from the PFT levels trend in a specific direction, as was seen in our patients. The distributional assumptions were checked before analyses.

## 3. Results

Over a four-month period during 2010-2011, we screened 44 patients and enrolled 40 patients (91% capture rate). Sixty-five percent of the patients were male, and the mean age was 8.7 years (SD 4.8). Fifty percent self-identified as African American, 25% were Caucasian, and 23% were Hispanic. We categorized the remaining 2% as “other,” due to the small number of participants. It is also noteworthy that approximately 30% of the patients were preschoolers (age < 5 years).

Fifty-five percent of the patients were classified as a mild-intermittent asthmatic, 30% were mild-persistent asthmatics, 12.5% were moderate-persistent asthmatics, and 2.5% were severe-persistent. This was a convenience sample in that coinvestigators were present in the ED based on scheduled research hours (4–8 hours a day Monday through Friday). The other four patients were not enrolled because nursing initiated treatment prior to performing PFTs. As stated above, PFTs were performed at baseline, prior to treatment with bronchodilators with or without oral corticosteroids. Patients who were unable to cooperate with the PFTs were not excluded unless no data were collected. Complete RIP data are available for 38 patients (95% compliance), and complete breathing pattern data are available for 37 patients (93% compliance).

Our 93% patient acceptance of the study and high compliance rate give insight to the feasibility of performing such a test in the ED. The majority of patients performed the assessment without difficulty. Patients as young as 3 years were able to perform the task with minimal coaching. The cases where RIP data were not obtained involved two patients, aged 3 and 14 years. The cases where data for the tidal breathing analysis were not obtained involved three patients, aged 17, 10, and 3 years. Age, therefore, was not a confounding factor in compliance. Once consent data were obtained, the initial assessment was performed in 15 minutes or less. The investigators did not have to discontinue any of the tests for patient safety reasons. The older and/or more cooperative patients had easier/shorter assessments.

**Figure 1:**
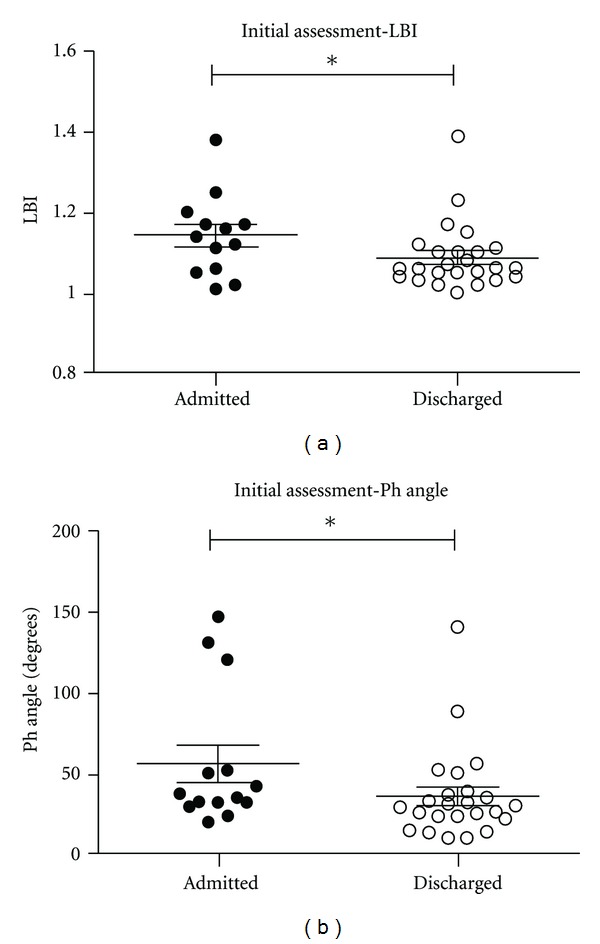
Summarized scatter plot results of (a) labored breathing index (LBI) and (b) phase angle (Ph angle) initial assessments as a function of admitted and discharged patients. Data are presented as mean ± SD. *P = 0.04 for 40 patients.

**Figure 2:**
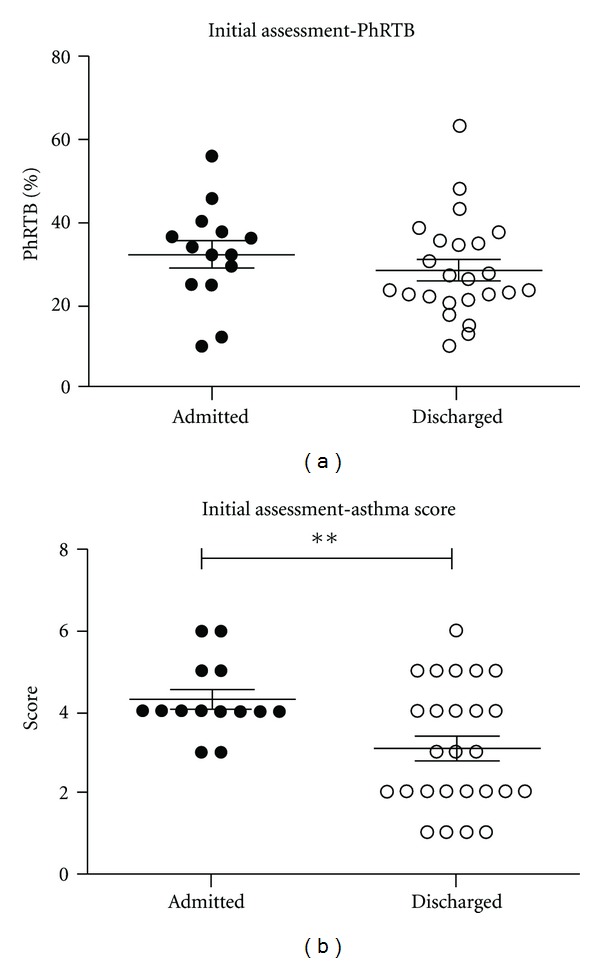
Summarized scatter plot results of (a) phase relation during total breath (PhRTB) and (b) asthma score initial assessments as a function of admitted and discharged patients. Data are presented as mean ± SD. **P < 0.01 for 40 patients.

The main outcome measure of the study was the association of PFT and patient disposition (admitted patients versus discharged patients). Demographic information, pertinent clinical information, and vital signs in the two groups are shown in Table 1. Means, standard deviations, and reference values for data obtained in PFTs are available in Table 2. PFT data are presented in box plot graphs (Figures 1 and 2(a)). Asthma score is also presented as a box plot graph (Figure 2(b)). 

The RIP results provided the following data: LBI (Figure 1(a)) was higher in the admitted versus discharged patients (*P* = 0.04). Phase angle (Figure 1(b)) showed a significant difference between the admitted and discharged patients (*P* = 0.04). Together, LBI and phase angle indicate that the TAA was increased in admitted patients. Phase relation (Figure 2(a)) results were not significantly different for the admitted versus discharged patients but did trend up in the admitted group. Compared to the predictive values for healthy children (Table 2), it can be demonstrated that our patients showed significant increases in TAA and thus represent patients presenting with obstructive respiratory disease [6, 12, 15].

Tidal breathing analysis data (Table 2) show that our patient population had a significantly lower tidal volumes (*P* < 0.001) compared with the predicted normal value. The *t*_PTEF_/*t*_*E*_ was similar between those patients admitted and discharged, but the value was significantly lower (*P* < 0.001) than the referenced control data [5]. This finding is indicative of the degree of obstruction in our population on presentation to the ED.

Finally, our study supports the strength of the asthma score (Figure 2(b)). Those admitted had a significantly higher (*P* < 0.01) asthma score (two-tailed test).

## 4. Discussion

In the United States, asthma accounts for nearly two million ED visits each year [1]. Management decisions are largely subjective and based on exam findings, vital signs, and pulse oximetry [2]. Symptoms are often underestimated by the child and parent. Children with longstanding symptoms are less likely to report symptoms and are more likely to present with hypoxia and a severe, life-threatening asthma exacerbation [3].

In the present pilot study, we demonstrated the feasibility and compliance of noninvasive PFT results in the ED, as well as the predictive value associated with disposition (admission versus discharge) to aid in resource utilization. Since these measurements have not been utilized in this setting, we set out to determine the feasibility of performing these measurements in this setting with the assistance of respiratory therapy, the compliance of patients with the evaluation techniques, and the post-hoc predictive value of the results (clinicians were initially blinded to the PFT results at the time of admission). Finally, we were interested in a convenient, noninvasive, assessment tool employing tidal breathing analysis that indicates the severity of the wheezing exacerbation. Thus, we are speculating that this objective diagnostic approach may be used in the future to determine from the moment of triage the likelihood that a given patient would require a prolonged ED visit. A decision regarding disposition could then be made early, decreasing the patient's ED length of stay (LOS). Our results supported our hypothesis in that we had a 93% patient acceptance of the study and high compliance rate, which supports the feasibility of performing such a test in the ED. Patients as young as 3 years were able to perform the task with minimal coaching, and age was not a confounding factor in compliance. Once consent data were obtained, the initial assessment was performed in 15 minutes or less.

Pulmonary function tests, including RIP and PT via a mask using a pediatric respiratory profile monitor, have been developed to evaluate patients who are minimally cooperative due to age (neonatal/pediatric population) or clinical condition as presented in the ED [5–10, 15, 16]. In earlier studies, these same PFT methods have been utilized in preterm and term infants, infants and children with skeletal dysplasias, and in children with asthma [9, 16–21]. In addition, the American Thoracic Society states that TAA and tidal expiratory flow analyses are promising techniques for assessing lung function in children [5]. Measurements are performed rapidly and repeatedly with minimum disturbance to the child, and there is potential for clinical use to assess patients in acute respiratory distress when other techniques cannot be applied [3].

The presence of TAA is reflective of increased work of breathing [5]. Normal reference values are available in healthy children [12]. In infants with airflow obstruction secondary to chronic lung disease or acute infection, the change in phase angle correlated with changes in lung resistance. In studies involving adolescents with cystic fibrosis and infants with bronchopulmonary dysplasia or bronchiolitis, the phase angle shift calculated from the motion indices of the rib cage versus the abdominal wall was significantly higher when compared to healthy controls [5]. Previous studies have also determined that changes measured by the RIP in infants with airflow obstruction after bronchodilators correlate well with changes in lung resistance and compliance [15]. These data validate the use of RIP in the evaluation and re-evaluation of asthmatics as an objective measure of the severity of asthma/wheezing exacerbation. 

As shown in the RIP data, we found that LBI, phase angle, and asthma scores were all significantly higher in those patients admitted versus discharged from the ED. It can be inferred that they had a more severe exacerbation that led to their required admission. Thus, prospective utilization of these parameters in the ED may provide an additional predictive value associated with disposition (admission versus discharge) to aid in resource utilization and to guide therapy. With a larger sample size, it may be possible to predetermine RIP values that further assist in predicting which patients require admission and the type of therapy required. Thus, increased predictability on the decision to admit greatly decreases LOS in the ED. Furthermore, patient satisfaction would likely increase if LOS in the ED were lessened.

**Figure 3:**
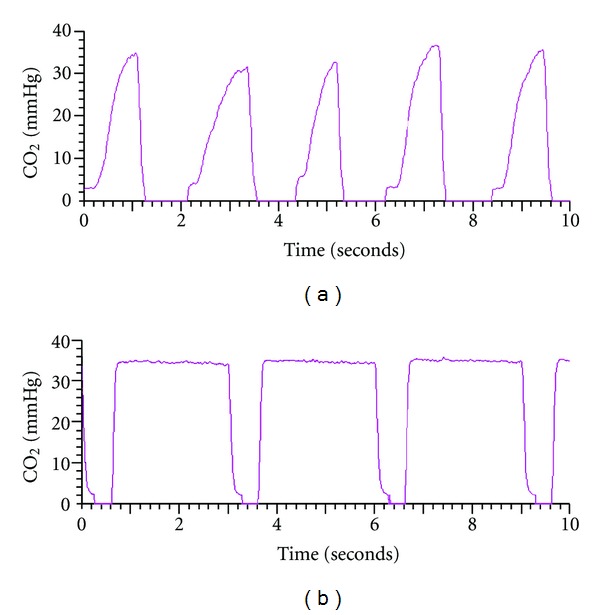
Pneumotachography measurements. (a) End-tidal CO_2_ pattern in an admitted 12-year-old patient enrolled in our study. Note the rise in end-tidal CO_2_ during the last portion of expiration (no clear plateau was observed). (b) End-tidal CO_2_ pattern of a control subject (from our pulmonary function test laboratory archives) demonstrating the plateau of normal CO_2_ waveform.

In addition to RIP data, the PT analysis provided pulmonary breathing patterns of tidal flow by simultaneous measurement of flow and volume and by CO_2_ analysis of expired gas. As noted in Figure 3, our patients typically demonstrated an uneven rise in end-tidal CO_2_ during expiration (compared to normal children). This rise in CO_2_ correlates with unequal emptying of CO_2_ from lung compartments, indicating lower-airway obstruction. Finally, the PFT equipment allows calculation of the above mentioned indices from flow and CO_2_ determinations, thus enabling real-time data collection and breath analysis for leaks and consistent tidal volume history.

There are currently no satisfactory normative data for tidal flow measurements in preschool children. Adults, children, and infants with wheezing disorders have been shown in most studies to have lower mean *t*_VEF_/*t*_*E*_ and *V*_PTEF_/*V*_*E*_ when compared to controls [5, 22]. In older children, *t*_VEF_/*t*_*E*_ has correlated well with FEV_1_. Treatment with bronchodilators in wheezing infants and young children has been shown to increase *t*_VEF_/*t*_*E*_. It has been reported that tidal breathing analysis is an objective measurement in asthmatics [3]. In this regard, tidal breathing analysis demonstrated that the patients enrolled in this study had a lower tidal volume compared to the predictive values. Furthermore, a larger sample size may have demonstrated that *t*_VEF_/*t*_*E*_ and *V*_PTEF_/*V*_*E*_ values correlate better with the current subjective measures for deciding patient admission versus discharge.

Our study had a few inherent limitations. Since the study was a pilot study, the initial results are based on a limited sample size. It is anticipated, however, that these positive outcomes, with regard to feasibility, test compliance, and potential predictability of admission, will enable us to perform a much larger prospective study in which PFT will be utilized in patient disposition and therapy guidance. Post-hoc analysis of the enrolled patients, again due to sample size, did not allow a uniform distribution of patients with regard to gender, age, race, or severity of disease. Furthermore, based on the observational study design, the PFT results could not be utilized to guide therapy or triage patients. With regard to PFT methodology, the RIP techniques have the advantage of being noninvasive, require no calibration or cooperation for phase and synchrony evaluations; however, accurate calibration of the RIP technology is difficult and requires detailed regression analysis using RIP and tidal breathing correlations [15]. In the present study, we used a noncalibrated RIP method for phase and synchrony evaluations and used an abbreviated two point RIP-tidal breathing calibration for evaluation of labored breathing index. Finally, it should be noted that the RIP Lissajous approach was developed for sinusoidal breathing patterns, and not complex patterns associated with some distressed breathing patterns. However, it has been shown that even in distorted loops, the calculated phase angle is still a good estimate of synchrony, and the error has been estimated at <10% [15].

## 5. Conclusions

Our data support the use of PFTs in the evaluation of asthmatics as an objective measure of the severity of the asthma/wheezing exacerbation in an ED setting. Noneffort-dependent PFTs can provide an objective value that can be used in the evaluation of an acute asthma exacerbation in the ED setting. These tests can be performed safely and efficiently in children with mild-to-moderate asthma on presentation to an ED. PFT results, like the clinical asthma score, are associated with patient disposition (admission versus discharge). As an objective measure of the acuity of an asthmatic patient, PFT results may better guide care, allowing an early decision on whether to admit a patient. This could decrease their LOS. Resource utilization and decreasing LOS in the ED are important in combating the national problem of ED overcrowding and can improve patient satisfaction. Finally, a more accurate sample size and power analysis can be calculated from our preliminary data for future prospective ED studies.

## Figures and Tables

**Table 1:** Summarized demographic data for 40 patients.

Demographics	Admitted	Discharged	*P* value*
Sex			
Female	4 (29%)	10 (71%)	0.5
Male	10 (39%)	16 (61%)	0.5
Race			
Caucasian	4 (40%)	6 (60%)	
African American	7 (35%)	13 (65%)	
Hispanic	2 (22%)	7 (78%)	
Other/unknown	1 (100%)	0 (0 %)	
Age (years)	7.4 (4.3)	9.4 (5.0)	0.2
Weight (kg)	32.5 (22.1)	47.3 (38.1)	0.2
Height (cm)	130 (29.9)	136.2 (27.2)	0.2
Length of stay (min)	326.85 (77.4)	221.96 (57.8)	0.07
Vital signs			
Heart rate	121.4 (18.8)	105.4 (20.9)	0.02
Respiratory rate	31.9 (9.2)	26.5 (8.1)	0.06
Pulse oximetry	97.2 (1.8)	97.8 (1.9)	0.3
Asthma score	4.3 (0.91)	3.1 (1.5)	0.01
Pertinent history			
Asthma/wheezing	14 (35%)	26 (65%)	0.7
Allergic rhinitis	10 (33%)	20 (67%)	0.7
Eczema	5 (42%)	7 (58%)	0.6
Exposure to smoke	4 (36%)	7 (64%)	0.9

Values represent number of patients (% of total) or mean (SD). ^∗^ *P* value for two-tailed *t*-test or chi-square.

**Table 2:** Summarized and statistical data for pulmonary function tests (PFT).

	Predicted mean (SEM)	Admitted (*n* = 14) mean (SD)	Discharged (*n* = 24) mean (SD)	^∗^ *P* value
LBI	1.01 (0.01)	1.27 (0.10)	1.08 (0.05)	0.04
Phase angle (°)	15.7 (4.0)	55.5 (43.1)	35.2 (28.3)	0.04
PhRTB (%)	10.1 (1.8)	32.4 (12.1)	28.5 (12.1)	0.17
Asthma score**	n/a	4.3 (0.9)	3.1 (1.5)	0.005

	Predicted average (Range)	Admitted (*n* = 14) mean (SD)	Discharged (*n* = 23) mean (SD)	^∗^ *P* value

ETCO_2 _(mmHG)	40 (35–45)	34.1 (4.5)	35.2 (3.7)	0.2
Tidal volume (mL/kg)	9 (7–10)	4.7 (1.8)^*ξ*^	4.6 (2.0)^*ξ*^	0.43
Minute ventilation (L)	7 (5–8)	3.9 (1.2)	4.1 (1.5)	0.3
*t*_PTEF_/*t*_*E*_	0.41 (0.05)	0.21 (0.09)	0.20 (0.08)	0.36
*V*_PTEF_/*V*_*E*_	n/a	1.01 (0.25)	1.0 (0.10)	0.44

LBI: labored breathing index; PhRTB: phase relation during total breath; ETCO_2_: end-tidal CO_2_; *t*_PTEF_: time to peak tidal expiratory flow; *t*_*E*_: total expiratory time; *V*_PTEF_: volume at peak tidal expiratory flow; *V*_*E*_: expired tidal volume. ^∗^ *P* value comparing the admitted versus discharged patients; one-tailed *t*-test. **P value comparing the admitted versus discharged patients; two-tailed *t*-test (*P* < 0.01). ^*ξ*^ *P* value comparing admitted and discharged patients to predicted controls; two-tailed *t*-test, (*P* < 0.001). Predicted values based on known healthy controls (12,15).
